# Prevalence of hepatitis B and D virus infection in a district of Mato Grosso, bordering Amazonas and Rondônia states

**DOI:** 10.1590/0037-8682-0559-2019

**Published:** 2020-10-21

**Authors:** Paulo Antonio, Elaine Cristina de-Oliveira, Thamires Oliveira Gasquez Martin, Eduardo Rodrigues, Lucas José da Silva, Francisco Campello do Amaral Mello, Cor Jesus Fernandes Fontes, Francisco José Dutra Souto

**Affiliations:** 1 Universidade Federal de Mato Grosso, Faculdade de Medicina, Hospital Universitário Júlio Müller, Cuiabá, MT, Brasil.; 2 Secretaria de Estado de Saúde de Mato Grosso, Superintendência de Vigilância em Saúde, Coordenação de Vigilância Epidemiológica, Cuiabá, MT, Brasil.; 3 Fundação Oswaldo Cruz, Instituto Oswaldo Cruz, Laboratório de Hepatites Virais, Rio de Janeiro, RJ, Brasil.

**Keywords:** hepatitis B, Prevalence, Hepatitis D, Amazon, Survey

## Abstract

**INTRODUCTION:**

Brazil’s western Amazon basin has the highest prevalence of hepatitis B virus (HBV) infection in the country. Coinfection with hepatitis D virus (HDV) is also endemic. To estimate the prevalence of HBV and HDV markers in a population inhabiting the northwest portion of Mato Grosso state in the western Amazon.

**METHODS:**

We performed a cross-sectional study of the seroprevalence of antibodies against HBV core antigen (anti-HBc) in the Três Fronteiras District northwest of Mato Grosso. Anti-HBc-positive subjects were tested for HBV surface antigen (HBsAg). Those positive for this marker were tested for HDV antibodies. Anti-HBc-negative participants were tested for anti-HBsAg. All tests were performed by EIA.

**RESULTS:**

A total of 623 individuals in the community were assessed; the majority (67.6%) were male, with a mean age of 30.8 ± 15.4 years. Two hundred and fourteen individuals (34.3%) were anti-HBc-positive, and 47 (7.5%) were HBsAg carriers. Only one individual was anti-HDV-positive. Among the 409 individuals without HBV infection, 18.3% were anti-HBsAg-positive. There was no association between HBV infection and known risk factors.

**CONCLUSIONS:**

The study area had intermediate-to-high endemicity for HBV infection, but a low prevalence of HDV. Our serological results suggesting low vaccination-induced protection indicate a need for reinforced immunization programs in the populations of northwest Mato Grosso.

## INTRODUCTION

The hepatitis B virus (HBV) is an important public health problem worldwide^1^. Complications of chronic HBV infection can lead to a significant disease burden with high morbidity and mortality rates due to cirrhosis or hepatocellular carcinoma. HBV infection occurs mainly in developing countries. In Brazil, the highest prevalence (as discovered at the end of the last century) is in the western Amazon, especially rural and remote areas^2^ ^,^ ^3^ ^,^ ^4^.

The hepatitis D or delta virus (HDV) is a replication defective agent that can only infect people in the presence of HBV^5^. Unlike HBV, HDV is restricted to certain geographical areas, such as the western Amazon. HDV superinfection in patients with chronic hepatitis B can produce acute liver failure with high mortality or concomitant chronic viral infection accelerating progression to cirrhosis^5^ ^,^ ^6^.

Mato Grosso is a geographically large state located in the center of Brazil. Its northwestern region corresponds to the southwest territory of the Amazon Basin, and borders the states of Rondônia and Amazonas, both of which have a high prevalence of HBV and HDV^7^ ^,^ ^8^. Since the 1990s, this region has become one of the last frontiers of human colonization in Brazil, although it has a floating population owing to a fluctuating local economy based on wood and mineral extraction. The human communities in this vast territory are scattered; they are far from large population centers, which renders access difficult. Sanitation conditions are precarious, and availability of basic health services is very limited. 

In the 1990s, a high prevalence of HBV infection was identified among the first displaced and settled populations in northwest Mato Grosso^9^ ^,^ ^10^. Additionally, an outbreak of hepatitis B occurred in one of these communities, although no specific risk factors were identified^11^. Gold miners were identified as a high-risk group for HBV at that time^11^ ^,^ ^12^. By contrast to the states of Amazonas, Acre, and Rondônia, there were no cases of HDV in northwest Mato Grosso^6^ ^,^ ^7^ ^,^ ^8^ ^,^ ^13^ ^,^ ^14^ ^,^ ^15^ ^,^ ^16^.

A new sero-epidemiological survey conducted in northwest Mato Grosso in 2001 indicated that the prevalence of HBV had decreased^17^. It also revealed that a significant portion of the population were immunized thanks to vaccination campaigns that had been undertaken after identification of high prevalence. By contrast to the findings of the previous studies, patients with HDV infections were identified this time, which was presumed to be due to increased immigration from the neighboring state of Rondônia.

More recent studies have shown a trend of declining HBV endemicity throughout Brazil^18^ ^,^ ^19^, which is attributed to infant vaccination programs established in the country approximately 20 years ago, and biosafety rules implemented in public and private institutions throughout the country (such as the requirement to use only disposable syringes and needles).

Using knowledge obtained through previous efforts to conduct a malaria study in a typical community in northwest Mato Grosso, we performed a cross-sectional study of markers of HBV and HDV infections. The objective was to estimate the prevalence of these two infections in this high-risk region, and assess the impact of vaccination programs on HBV infection endemicity. 

## METHODS

### Study area

This study was conducted in the Três Fronteiras District, located 400 km from the city of Colniza, Mato Grosso. The Três Fronteiras District borders three Amazon states, Mato Grosso, Rondônia, and Amazonas, and is located 1,530 km from the state capital, Cuiabá. In 2013, the community's population was estimated at approximately 1,300 people. Health services in the District are scarce; there is only one Primary Health Unit that performs nursing care, and microscopy examinations for malaria, and there are no local physicians. The district does not have paved roads, basic sanitation, electric lighting, or access to mobile phones. Moreover, access to the district is limited during the rainy season because of inability to maintain the roads linking it to neighboring communities. The community is divided into a main village, a settlement in the vicinity of the timber company, and another settlement in the mining sector. The region’s economy relies on cassiterite mining, gold mining (alluvial and mechanized), and logging. The Três Fronteiras District has an intense migratory flow between the three states driven by these economic activities, and many workers have temporary housing within the locality.

### Study design

The study consisted of a sero-epidemiological survey for markers of hepatitis B and D. Demographic and socioeconomic data, and risk factors for sexually and parenterally transmitted diseases were also collected and evaluated. All data and biological material collection occurred during July 2013.

Our intention was to include all individuals living in the Três Fronteiras district, since the population is not large. Thus, all individuals in the locality during the survey were invited to participate. All inhabited houses in the three main settlements were visited. People were approached in their homes and were presented with an informed consent form after its contents were explained. Any included minors had an adult family member approve their participation. Blood samples were processed, and serum aliquots stored at -20ºC in the bio-repository of the Júlio Muller University Hospital, Federal University of Mato Grosso.

### Laboratory analysis

Serological markers were assessed using enzyme immunoassays (ELISA). All samples were initially tested for the presence of immunoglobulin-G (IgG) antibodies against the core HBV antigen (anti-HBc) (Anti HBC Total, Symbiosis, ALKA, Brazil) at the Júlio Müller University Hospital laboratory. Positive samples were tested for HBV surface antigen (HBsAg) (HBsAg Autobio, ALKA, China) at the Central Laboratory of the State Department of Health of Mato Grosso. Samples that were negative for anti-HBc were tested in the same laboratory for antibodies against HBsAg (anti-HBs, DiaSorin, Brazil) with the aim of assessing vaccine coverage. The HBsAg-positive samples were tested for IgG antibodies against HDV (anti-HDV, Delta Total, DiaSorin, Brazil) at the Laboratory of Viral Hepatitis of the Oswaldo Cruz Institute, FIOCRUZ, Rio de Janeiro.

### Data analysis

Anti-HBc-positive individuals were considered to have been previously infected with HBV; those who were also positive for HBsAg were considered to be carriers. Anti-HBc-negative and anti-HBs-positive individuals were classified as immunized through vaccination. The demographic and epidemiological variables investigated were analyzed and compared to the results of the anti-HBc and HBsAg analyses to identify possible factors associated with HBV infection and carrier status, respectively.

A database was created using Excel 2013 software (Microsoft Corp., Redmond, WA, USA) and exported to EpiData Analysis 2.2 (http://www.epidata.dk; 2009) for descriptive statistics and bivariate analyses. Categorical variables are expressed as proportions, and continuous variables as means with standard deviations. The magnitudes of associations were estimated by calculating odds ratios and their respective 95% confidence intervals. Variables of interest with p values less than 10% were included in a multiple logistic regression model to detect potential confounding effects using Stata 11.0 statistical software (Stata Corp, College Station, USA, 2009). Significance for all analyses was set at 0.05. 

### Ethical Issues

This study was evaluated and approved by the Research Ethics Committee of the Júlio Müller University Hospital - Federal University of Mato Grosso, under protocol no. 555.706, as an amendment to protocol no. 158.109. 

## RESULTS

A total of 623 individuals (between 40% and 50% of the estimated community) from 125 households in the district of Três Fronteiras were included in the study. Refusals were rare. The sample comprised 313 individuals from the main village, 280 living in the vicinity of the timber company, and 30 the mining sector’s inhabitants. People living remote from the main villages or traveling during the visits were not surveyed. The households were fragile dwellings constructed using wood, canvas, or masonry without plaster. The subjects did not have access to safe drinking water or garbage collection, and there was no sewage system, although rudimentary septic tanks were present in a few households. 

Of the individuals included in the study, 202 (32.4%) were female. The mean age was 30.8 ± 15.4 years. Most of the individuals (64.5%) were between 20 and 49 years of age. The majority had only basic education (71.6%) and were brown (59.1%). Other participant demographics are shown in Table 1.

**TABLE 1:** Demographic characteristics and health information of participants in the survey of hepatitis B markers in Três Fronteiras, Colniza, Mato Grosso, Brazil.

Variables	N (%)
Total number of participants	623 (100.0)
Age (years)	
Mean ± SD	30.8±15.4
Median [2nd, 3rd quartile]	31 [20, 43]
Extremes	4 months - 85 years
Age groups (years)	
0-9	60 (9.6)
10-19	90 (14.4)
20-29	139 (22.3)
30-39	149 (23.9)
40-49	114 (18.3)
≥50	71 (11.4)
Sex	
Male	421 (67.6)
Female	202 (32.4)
Skin color	
White	148 (23.8)
Black	105 (16.8)
Brown	368 (59.1)
Yellow	2 (0.3)
Schooling	
None	60 (9.6)
Elementary	446 (71.6)
Secondary	101 (16.2)
Higher education	16 (2.6)
Locality	
Village	313 (50.3)
Logging	280 (44.9)
Gold mining	30 (4.8)
Have you ever had jaundice?	
Yes	5 (0.8)
No	618 (99.2)
Have you ever had hepatitis?	
Yes	10 (1.8)
No	613 (98.4)
Hepatitis B vaccine	
Yes	169 (27.1)
No	454 (72.9)
Have you ever lived in the north region?	
Yes	563 (90.4)
No	60 (9.6)
Have you ever been hospitalized?	
Yes	132 (21.2)
No	491 (78.8)
Have you ever been transfused?	
Yes	11 (0.3)
No	612 (99.7)
Have you ever undergone surgery?	
Yes	44 (7.1)
No	579 (92.9)
Are you involved in gold mining?	
Yes	30 (4.8)
No	593 (95.2)

Two hundred and fourteen individuals (34.3%) had previously been infected with HBV, and 47 (7.5%) were carriers of the virus. One 59-year-old man among the 47 individuals with HBV was positive for anti-HDV IgG (2.1%) (Table 2).

**TABLE 2:** Assessment for markers of hepatitis B and D in Três Fronteiras, Colniza, Mato Grosso, Brazil.

Total number of participants	623 (100.0)
Infection by HBV (anti-HBc/total)	
Yes	214 (34.3)
No	409 (65.7)
HBV carriers (HBsAg/total)	
Yes	47 (7.5)
No	576 (92.5)
HBV carriers among exposed (HBsAg/anti-HBc)	
Yes	47 (21.9)
No	167 (78.1)
HBV carriers exposed to HDV (anti-HDV IgG)	
Yes	1 (2.1)
No	46 (97.9)
Vaccination profile among those not exposed to HBV (anti-HBsAg alone)	
Yes	75 (18.3)
No	334 (81.7)

**Anti-HBc:** hepatitis B core antigen antibodies; **HBsAg:** hepatitis B surface antigen; **HBV:** hepatitis B virus; **HDV:** hepatitis D virus; **IgG:** immunoglobulin G.

No association was found between exposure to HBV and skin color, schooling, history of jaundice or hepatitis, hospitalization, blood transfusion or surgery, dwelling, alcohol consumption, or having lived in the northern region of the country. However, male sex and older age were associated with anti-HBc positivity. In multivariate analysis, only age remained a significant factor, especially the 30-39 (P=0.041) and ≥50 year (P=0.015) old cohorts. (Table 3).

**TABLE 3:** Univariate and adjusted analysis of HBV (anti-HBc positive) infection according to demographic variables and potential risk factors in Três Fronteiras, Colniza, Mato Grosso, Brazil, in 2013.

Variables	Anti-HBc		OR	OR	OR*	OR
	Positive (%)	Negative (%)		95% CI		95% CI
Sex						
Female	56 (27.7)	146 (72.3)	1.0		1.0	
Male	158 (37.5)	263 (62.5)	1.5	1.1-2.2	1.4	1.0-2.1
Age range (years)						
0-9	13 (21.7)	47 (78.3)	1.0**		1.0	
10-19	20 (22.2)	70 (77.8)	1.03	0.4-2.9	1.0	0.5-2.2
20-29	51 (36.7)	88 (63.3)	2.1	0.8-5.3	1.9	0.9-4.0
30-39	57 (38.3)	92 (61.7)	2.2	0.9-5.6	2.1	1.0-4.2
40-49	42 (36.8)	72 (63.2)	2.1	0.8-5.4	1.9	0.9-4.1
≥50	31 (43.7)	40 (56.3)	2.8	1.0-7.7	2.6	1.2-5.8
Skin color						
White	43 (29.1)	105 (70.9)	1.0^#^		1.0	
Black	42 (40.0)	63 (60.0)	1.6	0.8-3.2	1.5	0.9-2.6
Brown	129 (35.1)	239 (64.9)	1.3	0.8-2.3	1.32	0.9-2.0
Yellow	0 (0)	2 (100.0)	-			-
Schooling						
None	18 (30.0)	42 (70.0)	1.0^#^			
Elementary	162 (36.3)	284 (63.7)	1.3	0.6-2.9		
Secondary	30 (29.7)	71 (70.3)	1.0	0.4-2.5		
Higher education	4 (25.0)	12 (75.0)	0.8	0.1-4.1		
History of jaundice						
No	213 (34.5)	405 (65.5)	1.0			
Yes	1 (20.0)	4 (80.0)	0.48	0.05-4.3		
Hospitalization						
No	170 (34.6)	321 (65.4)	1.0			
Yes	44 (33.3)	88 (66.7)	0.4	0.6-1.4		
Blood transfusion						
No	212 (34.6)	400 (65.4)	1.0			
Yes	2 (18.2)	9 (81.8)	0.4	0.1-1.9		
Surgery						
No	202 (34.9)	377 (65.1)	1.0			
Yes	12 (27.3)	32 (72.7)	0.7	0.3-1.4		
Anti-HBV vaccine						
No	158 (34.8)	296 (65.2)	1.0	0.6-1.3		
Yes	56 (33.1)	113 (66.9)	0.9			
History of hepatitis						
No	211 (34.4)	402 (65.6)	1.0			
Yes	3 (30.0)	7 (70.0)	0.8	0.2-3.2		
Alcohol consumption						
No	163 (36.1)	289 (63.9)	1.0			
Yes	35 (31.8)	75 (68.2)	0.7	1.1-2.8		
Lived in the northern region						
No	24 (40.0)	36 (60.0)	1.0			
Yes	190 (33.7)	373 (66.3)	0.8	0.4-1.3		
Gold mining						
No	13 (43.3)	17 (56.7)	1.0			
Yes	201 (33.9)	392 (66.1)	1.5	0.7-3.1		

*Adjusted OR in a logistic regression model (pseudo-R^2^: 0.027, P=0.01). **Chi-square for trend. ^#^used as reference layer. **CI**: confidence interval; **HBcAg:** hepatitis B core antigen; **HBV:** hepatitis B virus; **OR:** odds ratio.

HBsAg-positivity was associated with male gender (9.0% vs. 4.5%, p = 0.043) in crude analysis, but the association was lost after adjusting for sex, age, and other variables (p = 0.107). Multivariate analysis revealed inverse associations between HBV carrier status (HBsAg positivity) and having been vaccinated (P=0.004) or living in the northern region (P=0.038), although the logistic regression model was not very explanatory (pseudo-R^2^ = 0.027) (data not shown). 

Among the 409 non-HBV-infected individuals, 75 (18.3%) had a profile indicating vaccine immunization (anti-HBs alone). The vaccine protected profile was low across all age groups, including children (Table 4). 

**TABLE 4:** Serological pattern of vaccine protection against HBV (anti-HBsAg positivity) among uninfected individuals in Três Fronteiras, Colniza, Mato Grosso, Brazil, 2013.

Variables	Anti-HBsAg	
	Positive (%)	Negative (%)
Age range (years)		
0-9	5 (10.6)	45 (89.4)
10-19	10 (14.3)	60 (85.7)
20-29	18 (20.4)	70 (89.6)
30-39	18 (19.6)	74 (80.4)
40-49	14 (19.4)	58 (80.6)
≥50	10 (25.0)	30 (75.0)
Total	75 (18.3)	334 (80.7)

**HBV:** hepatitis B virus; **HBsAg:** hepatitis B surface antigen.

## DISCUSSION

This survey focused on HBV and HDV prevalence in a west Amazonian population comprised of low-income individuals with low education levels, most of whom had migrated from other areas of the Amazon. Approximately one-third of the assessed individuals had previously been infected by HBV, and 7.5% were HBsAg-positive, indicating moderate-to-high endemicity for HBV. These figures differ from those reported in national and regional studies that showed a fall in endemicity throughout Brazil, including in the Amazon region^18^ ^,^ ^19^.

The population has settled this area for the purposes of logging and mining. The distance from more developed urban centers, combined with the challenges inherent to living in a tropical forest and the nature of their jobs renders these individuals vulnerable to violence, trauma, and tropical diseases. The lack of sanitary conditions and assistance from health authorities or public security officials contributes to this vulnerability. Communities living under such conditions in northwest Mato Grosso in the 1990s were found to have a high HBV infection prevalence^1^ ^,^ ^12^. At that time, the government accelerated anti-HBV vaccination efforts, resulting in a decrease in the disease’s prevalence in subsequent years^12^. However, the region continued to attract low-income, poorly educated people. Although HBV vaccination became mandatory for children in Brazil in the late 1990s, follow up assessments of HBV endemicity have been scarce over the last 15 years. 

A national survey promoted by the Brazilian Ministry of Health over the last decade revealed a low prevalence of the disease across all regions of the country, although only large urban centers were surveyed^18^. Historically, however, the highest prevalence of the disease has always been in rural regions and smaller cities. The results of the present study suggest that HBV circulation is still pronounced in the most remote regions of the Amazon, where basic services are lacking.

Our analysis of demographic data revealed that low education levels, having lived in a high-risk area such as the northern region of Brazil or in a mining community, and HBV-specific risk factors such as blood transfusions did not reveal any primary route of infection that would explain the high HBV prevalence. This suggests that alternative modes of infection took place in the community, including sexual or other horizontal modes of transmission. Only increasing age was associated with higher frequency of HBV markers, likely owing to longer duration of exposure. Details of sexual practices or use of illegal drugs could not be obtained, given the conditions in which the interviews were conducted.

The high proportion of HBV carriers relative to the total number of previously infected people (21.9%) is noteworthy. Generally, HBV carriers comprise up to 10% of individuals who have been exposed to the agent in areas of high prevalence. Exceptions are observed in areas where HBV circulation is very prevalent or has subsided only recently^12^ ^,^ ^20^. To determine if HBV carriers were infected recently, it would be necessary to test anti-HBc immunoglobulin-M (IgM) fractions, which was beyond the scope of our study.

The low vaccination coverage in our population is also a cause for concern. Only 27% of surveyed individuals remembered having been vaccinated, and the serological marker indicative of vaccine protection was identified in only 18% of uninfected individuals, consistent with the information provided by the participants. A previous study in a small city in a non-Amazonian region in Mato Grosso revealed that 56% of assessed adolescents exhibited evidence of a vaccine response (anti-HBsAg-positivity alone)^21^. Data from our present study are more alarming given that vaccination-related serological patterns were even worse in individuals under 20 years of age. Intensifying vaccination efforts is essential, especially among young people, since two-thirds of the community comprises susceptible individuals in whom the spread of HBV is more likely (as has been observed in similar communities in the Amazon).

As the vast majority (90.4%) of individuals in our study came from the northern region of Brazil, where high HBV and HDV prevalence is expected, delta superinfection was an important concern. Fortunately, however, only one individual was found to have been infected by both viruses. It is nonetheless necessary to maintain surveillance for HDV, since large numbers of chronic carriers generate conditions favorable to dissemination of this agent if introduced into the region. 

Our survey has some limitations. One limitation was the lack of funding, which prevented testing participants for a wider array of informative serological markers. Thus, we were unable to determine whether there were additional anti-HDV-positive subjects among the HBsAg-negative and anti-HBc-positive individuals. Anti-HBc IgM assessment in HBsAg-positive individuals, which would have revealed whether HBV circulation began recently in the community, was also not possible. An important limitation was that only roughly half of the estimated local population was sampled. Demographic data from the settlements rapidly become inaccurate due to the migratory lifestyles of the individuals, and lack of official information from the municipality during the survey. Two thirds of the sample population were men, which could represent a selection bias. However, wood and mineral extraction, the main labor activity practiced in these settlements, usually generates a predominantly male community. In any case, male gender is a known risk factor for HBV infection. In fact, HBsAg-positivity was twice as high among men as among women in our sample. This factor may have increased the overall prevalence of HBV markers in the sample. Another limiting aspect was that some potential risk behavior variables could not be assessed due to lack of privacy during the interviews and completion of questionnaires in some households. This may have contributed to lack of sufficient power to assess significance in multivariate analyses. 

In summary, some communities in Mato Grosso retain high proportions of HBV carriers, and even higher numbers of susceptible individuals. These characteristics indicate the need to resume vaccination efforts for the entire community, especially the young. Our data also revealed that HDV is not an immediate concern in this community.

## References

[1] World Health Organization (WHO) (2017). Global Hepatitis Report 2017.

[2] Bensabath G, Boshell J (1973). Presença do antígeno Austrália em populações do interior do Estado do Amazonas - Brasil. Rev Inst Med Trop Sao Paulo.

[3] Fonseca JC, Simonetti SR, Schatzmair HG, Castejón MJ, Cesário AL, Simonetti P (1988). Prevalence of infection with hepatitis delta virus among carriers of hepatitis B surface antigen in Amazonas State, Brazil. Trans Royal Soc Trop Med Hyg.

[4] Souto FJD (1999). Distribuição da hepatite B no Brasil: atualização do mapa epidemiológico e proposições para seu controle. Gastroenterol Endosc Dig.

[5] Noureddin M, Gish R (2014). Hepatitis delta: epidemiology, diagnosis and management 36 years after discovery. Curr Gastroenterol Rep.

[6] Braga WS, Castilho M da C, Borges FG, Leão JR, Martinho AC, Rodrigues IS, Azevedo EP, Barros GM, Paraná R (2012). Hepatitis D virus infection in the Western Brazilian Amazon - far from a vanishing disease. Rev Soc Bras Med Trop.

[7] Vieira GB, Florão M, Castro KP, Alves TC, Vaiciunas S, Honda ER, Camargo LM, de Sousa CM (2015). Hepatitis B in Rondônia (western Amazon region, Brazil): descriptive analysis and spatial distribution. Arq Gastroenterol.

[8] Botelho-Souza LF, Vasconcelos MPA, Dos Santos AO, Salcedo JMV, Vieira DS (2017). Hepatitis delta: virological and clinical aspects. Virol J.

[9] Souto FJ, Fontes CJ, Gaspar AM, Paraná R, Lyra LG (1996). Concomitant high prevalence of hepatitis C virus antibodies and hepatitis B virus markers in a small village of the Amazon region. Rev Inst Med Trop Sao Paulo.

[10] Souto FJ, Fontes CJ, Gaspar AM, Lyra LG (1998). Hepatitis B virus Infection in immigrants to the Southern Brazilian Amazon. Trans Royal Soc Trop Med Hyg.

[11] Souto FJD, Fontes CJF, Gaspar AMC (1998). Outbreak of hepatitis B virus in recent arrivals to the Brazilian Amazon. J Med Virol.

[12] Souto FJD, Fontes CJF, Gaspar AMC (2001). Prevalence of hepatitis B and C virus markers among malaria-exposed gold miners in Brazilian Amazon. Mem Inst Oswaldo Cruz.

[13] Viana S, Paraná R, Moreira RC, Compri AP, Macedo V (2005). High prevalence of hepatitis B virus and hepatitis D virus in the western Brazilian Amazon. Am J Trop Med Hyg.

[14] Paraná R, Kay A Molinet F, Viana S, Silva LK, Salcedo J, Tavares-Neto J, Lobato C, Rios-Leite M, Matteoni L, OliveiraJr A, Tauil P, Trépo C (2006). HDV genotypes in the Western Brazilian Amazon region: a preliminary report. Am J Trop Med Hyg.

[15] Katsuragawa TH, de Almeida Cunha RP, Salcedo JMV, de Souza DCA, de Oliveira KRV, Gil LHS, Batista DP, Tada MS, da Silva LHP (2010). Alta soroprevalência dos marcadores das hepatites B e C na região do alto rio Madeira, Porto Velho, Rondônia, Brasil. Rev Pan-Amaz Saude.

[16] Silva ACB, Souza LFB, Katsuragawa TH, de Lima AA, Vieira DS, Salcedo JMV (2015). Perfil soroepidemiológico da hepatite B em localidades ribeirinhas do rio Madeira, em Porto Velho, Estado de Rondônia, Brasil. Rev Pan-Amaz Saude.

[17] Souto FJ, Fontes CJ, Oliveira SS, Yonamine F, Santos DR, Gaspar AM (2004). Prevalência da hepatite B em região rural de município hiperendêmico na Amazônia Mato-grossense: situação epidemiológica. Epidemiol Serv Saude.

[18] Pereira LM, Martelli CM, Hamann EM, Montarroyos UR, Braga MC, Lima ML, Cardoso MR, Turchi MD, Costa MA, de Alencar LC, Moreira RC, Figueiredo GM, Ximenes RA (2009). Hepatitis Study Group. Population-based multicentric survey of hepatitis B infection and risk factor differences among three regions in Brazil. Am J Trop Med Hyg.

[19] Souto FJD (2016). Distribution of hepatitis B infection in Brazil: the epidemiological situation at the beginning of the 21st century. Rev Soc Bras Med Trop.

[20] Braga WS, Brasil LM, Souza RA, Castilho MC, Fonseca JC (2001). The occurrence of hepatitis B and delta virus infection within seven Amerindian ethnic groups in the Brazilian western Amazon. Rev Soc Bras Med Trop.

[21] Melo LV, Silva MA, Calçada CO, Cavalcante SR, Souto FJ (2011). Hepatitis B vírus markers among teenagers in the Araguaia region, Central Brazil: assessment of prevalence and vaccination cover. Vaccine.

